# Dissecting the impact of target-binding kinetics of protein binders on tumor localization

**DOI:** 10.1016/j.isci.2021.102104

**Published:** 2021-01-29

**Authors:** Yunjin Song, Hoibin Jeong, Song-Rae Kim, Yiseul Ryu, Jonghwi Baek, Jinhak Kwon, Hyeongjun Cho, Kil-Nam Kim, Joong-jae Lee

**Affiliations:** 1 Department of Biochemistry, Kangwon National University, Chuncheon 24341, South Korea; 2 Chuncheon Center, Korea Basic Science Institute (KBSI), Chuncheon 24341, South Korea; 3 Institute of Life Sciences (ILS), Kangwon National University, Chuncheon 24341, South Korea; 4 Global/Gangwon Innovative Biologics-Regional Leading Research Center (GIB-RLRC), Kangwon National University, Chuncheon 24341, South Korea

**Keywords:** biochemistry, molecular medicine, immunology

## Abstract

Systematic control of *in vivo* behavior of protein-based therapeutics is considered highly desirable for improving their clinical outcomes. Modulation of biochemical properties including molecular weight, surface charge, and binding affinity has thus been suggested to enhance their therapeutic effects. However, establishing a relationship between the binding affinity and tumor localization remains a debated issue. Here we investigate the influence of the binding affinity of proteins on tumor localization by using four repebodies having different affinities to EGFR. Biochemical analysis and molecular imaging provided direct evidence that optimal affinity with balanced target binding and dissociation can facilitate deep penetration and accumulation of protein binders in tumors by overcoming the binding-site-barrier effect. Our findings suggest that binding kinetics-based protein design can be implicated in the development of fine-tuned protein therapeutics for cancers.

## Highlights

• High binding affinity limits the tumor localization of protein binders *in vivo*
• Moderate-affinity binders can exhibit better tumor localization than higher binders
• Binding kinetics of binders play a central role in controlling tumor localization
• Exploring the optimal affinity of binders can enhance their therapeutic potential

## Graphical Abstract

**Figure:**
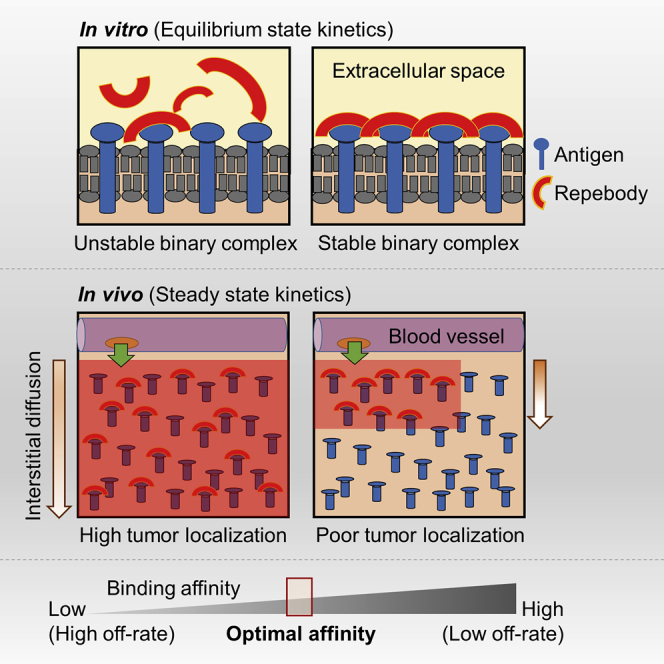

## Teaser

Biochemistry; molecular medicine; cancer

## Introduction

Biopharmaceuticals or biologic drugs (biologics) are an attractive therapeutic option for treating cancer owing to their profound efficacy (Ghilardi et al., 2020; Nabhan et al., 2018; Khalil et al., 2016). In most cases, biologics have higher efficacy and lower systemic adverse effects than traditional chemical drugs (Schirrmacher, 2018). The highly ordered three-dimensional structures of biologics enable them to have exceptional target selectivity and affinity than their traditional counterparts (Kintzing et al., 2016). Biologics comprise a wide range of biologically derived functional substances, including peptides, proteins, antibodies, nucleotides, and cell-based products (Khalil et al., 2016; Morrow and Felcone, 2004; Roberts et al., 2020). Of these, the protein-based drugs have recently emerged as the fastest growing class of biologics in targeted therapy, because of the remarkable clinical outcomes (Lagassé et al., 2017). Particularly, monoclonal antibodies are by far the largest and most promising biologics in malignant tumors and autoimmune diseases, constituting about seven of the top ten global pharmaceutical products (Urquhart, 2020).

**Figure 1:**
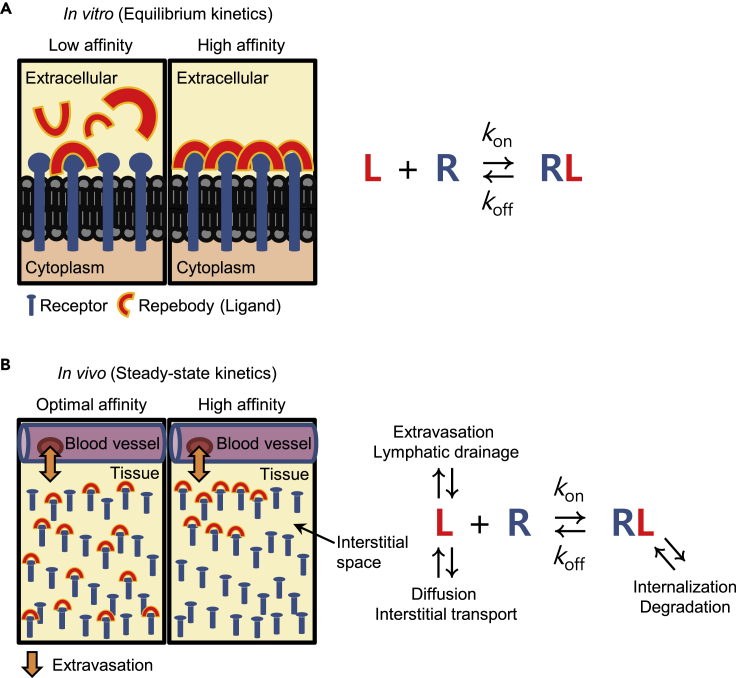
A schematic representation of the *in vitro* and *in vivo* tumor localization of repebodies with relation to the binding affinities (A) *In vitro* binding of EGFR-specific repebodies to an antigen occurs in proportion to the binding affinities (left). The *in vitro* environment is similar to a closed system. The ligand-receptor binding reactions will thus reach equilibrium after sufficient time has elapsed, which can be explained by the Langmuir adsorption model (right). In this state, repebodies with high affinity show a more tight binding to the receptors than the ones with low affinity. (B) *In vivo* environment is considered as an open system in which kinetics of free and bound ligands can be dynamically affected by various physiological processes (right). When repebodies were transported into tumors via extravasation, almost all high-affinity repebodies tend to tightly bind to the first encountered perivascular receptors with low dissociation. The formation of stable receptor-ligand binary complexes progressively inhibits the accumulation of repebodies in deeper regions of the tumor (left). On the other side, repebodies having an optimal affinity can penetrate deeply into the tumor in an unbound form via preferential diffusion and interstitial transport, which result from a relatively high off-rate (*k*_off_).

Biochemical factors of proteins such as surface charge, molecular size, and binding affinity have an effect on their biological properties (Boswell et al., 2010; Holliger and Hudson, 2005; Kuna et al., 2018; Rudnick et al., 2011). In drug discovery and development, these factors are considered to be closely related to pharmacokinetics, tissue penetration, and distribution of protein-based drugs, all of which affect efficacy and safety (Thurber et al., 2008a, 2008b). Therefore, systemic evaluation of the relationship between biochemical properties and *in vivo* behavior is essential to improve the effectiveness of protein-based therapeutics. Transvascular transportation has been thoroughly investigated to determine the effects of molecular weight and size of macromolecules on tumor penetration by showing that the transport rates are inversely proportional to their molecular sizes (Jain, 1990; Yuan et al., 1995; Wirthl et al., 2020). Furthermore, previous studies using antibodies and scaffold proteins have also demonstrated that *in vivo* tumor localization of the protein is restricted to its molecular weight due to limited extravasation and interstitial diffusion (Yokota et al., 1992; Debie et al., 2020; Nessler et al., 2020). In general, the ability of protein-based drugs to penetrate tumor tissues is believed to be proportional to their binding affinity (Figure 1A) (Carter, 2001). Designed ankyrin repeat protein (DARPin; ∼15 kDa) typically showed a positive correlation between binding affinity and tumor targeting (Zahnd et al., 2010). The observed *in vivo* phenomenon was well agreed with computational analysis concerning the impact of molecular size and binding affinity on tumor uptake (Schmidt and Wittrup, 2009). The studies indicated that higher binding affinity is required for very small proteins to prevent their rapid elimination in the bloodstream and the extracellular fluid and to be retained in tumors. On the other hand, several reports demonstrated that the binding strength of protein binders including single-chain variable fragments (scFvs; ∼28 kDa), dimeric nanobodies (∼30 kDa), and antibodies (∼150 kDa) does not always correlate with their accumulation in tumors (Rudnick et al., 2011; Debie et al., 2020; Adams et al., 2001; Saga et al., 1995; Tsumura et al., 2018). High-affinity binders tend to be preferentially accumulated around blood vessels in tumor tissues, physically restricting subsequent extravasation and tumor penetration (Figure 1B). This phenomenon is referred to as the binding-site barrier effect (Saga et al., 1995), implying that an optimal binding affinity might be required to maximize transport efficiency of proteins inside tumors. In addition, proteins larger than the size cutoff for glomerular filtration are capable of high tumor localization even with relatively low binding affinity, which is likely to be the result of the binding-site barrier effect and their long circulation half-life. Taken together, previous results have not been able to clearly establish the relationship between binding affinity and tumor localization *in vivo*. Considering that small changes in biochemical properties can lead to significant clinical outcomes, studying the impact of binding affinity on tumor localization can guide in the effortless development of efficacious protein therapeutics.

A repebody is a small (∼30 kDa) non-immunoglobulin, newly designed scaffold based on the variable lymphocyte receptors of jawless vertebrates (Lee et al., 2012). By phage display selection and stepwise modular engineering, various repebodies have been successfully developed with high affinity and selectivity for disease-related targets (Son et al., 2020; Sohn et al., 2020; Sohn and Kim, 2020; Duarte et al., 2020; Seo et al., 2017; Kim et al., 2016; Hwang et al., 2016a, 2016b, 2016c; Lee et al., 2014, 2015; Heu et al., 2014). The human epidermal growth factor receptor (EGFR)-specific repebody exemplifies such a scaffold where the robust targeting moiety has been extensively exploited for targeted therapy and diagnosis with negligible toxicity (Lee et al., 2015, 2017; Ryu et al., 2018, 2020; Yun et al., 2017). Despite significant progress and widespread applications, the *in vivo* tumor localization of repebodies related to binding affinity and kinetics has not yet been studied systematically. The present study investigated the *in vivo* tumor accumulation of four EGFR-specific repebodies with different binding affinity (*K*_D_ ranged from 14 nM to 51 pM) by the *in vivo* near-infrared (NIR) fluorescence imaging in xenograft mouse models. Cell-based and biochemical assays showed that the *in vitro* targeting ability of repebodies is highly correlated with their binding affinity. Contrary to *in vitro* results, it was demonstrated that an optimal level of binding kinetics and affinity can give rise to the highest tumor localization of repebodies by overcoming the binding-site barrier. Details are reported herein.

## Results

### Biochemical evaluation of EGFR-specific repebodies

**Figure 2:**
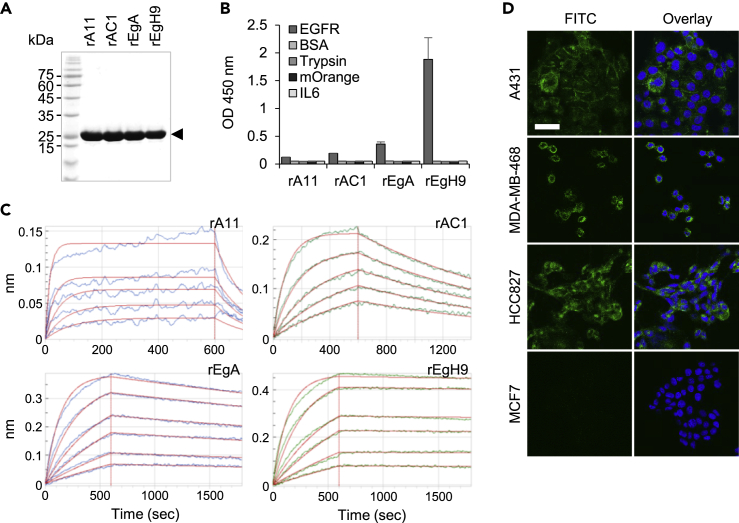
Bacterial production and characterization of EGFR-specific repebodies (A) SDS-PAGE analysis of four different repebodies (28 kDa) expressed in *E. coli*. After conducting size-exclusion chromatography, the purity of all eluted repebodies was determined to be 95%. (B) ELISA analysis for evaluating the specific binding property of repebodies against EGFR. All antigens (EGFR, bovine serum albumin [BSA], Trypsin, mOrange, and IL-6) were coated at a concentration of 10 μg/mL. BSA was used as a negative control. Error bars indicate standard deviations of triplicate experiments. (C) Binding kinetics assay of repebodies for EGFR was based on the Octet system. Based on measured sensorgrams, the equilibrium dissociation constants (*K*_D_) and rate constants (*k*_on_ and *k*_off_) were estimated, as described in Table 1. (D) Fluorescence cell imaging for identifying specific binding of repebodies rEgH9 using various cancer cell lines. The cancer cells were treated with fluorescein-labeled repebodies (at a concentration of 10 μg/mL). The A431, MDA-MB-468, and HCC827 cell lines exhibited high levels of EGFR expression, whereas the MCF7 cells expressed a low level of EGFR (Figure S4). Treated cells were visualized by confocal microscopy. The nuclei were stained with Hoechst 33342 (blue). Scale bar, 50 μm.

Human EGFR-specific repebodies have been previously developed through phage display and modular evolution (Lee et al., 2015). Four different repebodies, namely, rA11, rAC1, rEgA, and rEgH9, have gradually increased binding affinities and share the same epitope for EGFR. All the constructs were expressed in a soluble form in bacteria, each yielding about 50 mg/L of culture. The repebodies were isolated in a highly purified form through affinity chromatography and subsequently purified through gel permeation chromatography. All the monomeric repebodies were eluted as a single major peak at about 70 mL of the elution volume (Figure S1). The SDS-PAGE analysis showed the purified repebodies to have a molecular weight of 28 kDa and purity greater than 95% (Figure 2A).

Biotinylated repebodies were subjected to an enzyme-linked immunosorbent assay to evaluate target specificity and relative binding affinity. All the repebodies exhibited highly specific binding signals for EGFR but negligible binding signals for the other control proteins (Figure 2B). Interestingly, the EGFR-binding signals were found to gradually increase in an affinity-dependent manner. Although previous biochemical studies have compared the relative binding affinities of repebodies (Lee et al., 2015), these parameters have not been estimated for the four EGFR-specific repebodies used in the present study. The equilibrium dissociation constants (*K*_D_) and kinetic rate constants of all the EGFR-specific repebodies were determined using the Octet analysis. The repebodies, rAC1, rEgA, and rEgH9 exhibited a 4-, 30-, and 285-fold increase in affinity compared with the initial binder, rA11 (Figures 2C and Table 1). The observation that the repebodies (rAC1, rEgA, and rEgH9) with improved affinities have a similar association rate constant (*k*_on_) justified the dissociation rate constant (*k*_off_) as the main driving force for increased binding affinity. The group of repebodies with a broad range of affinities were expected to be ideal for investigating the affinity-based differences in tumor localization (with the uncontrolled variables excluded) because they shared a strong sequence identity (>95%) (Lee et al., 2015) and bound a common epitope on EGFR (Figure S2).

**Table 1:** Binding affinity and kinetic analysis of EGFR-specific repebodies

Repebody	Octet analysis^a^				FACS^b^
	*k*_on_ (M^−1^s^−1^)^c^	*k*_off_ (s^−1^)^d^	*K*_D_ (M)^e^	*t*_1/2_ (min)^f^	*K*_1/2_ (M)^g^
rA11	5.42×10^5^	7.83×10^−3^	1.44×10^−8^	1.50	1.51×10^−6^
rAC1	2.06×10^5^	7.16×10^−4^	3.48×10^−9^	16.1	1.86×10^−7^
rEgA	2.92×10^5^	1.37×10^−4^	4.70×10^−10^	84.3	2.56×10^−8^
rEgH9	2.95×10^5^	1.49×10^−5^	5.05×10^−11^	775	2.21×10^−9^

a The Octet data showed slightly different binding affinity compared with previously obtained isothermal titration calorimetry (ITC) data (Lee et al., 2015), resulting from differences in measurement methods (ITC is a solution-based technique, but Octet analysis is performed on an antigen-immobilized surface).

b All FACS data were generated by using Cy 5.5-conjugated repebodies.

c Association rate constant.

d Dissociation rate constant.

e Equilibrium dissociation constant.

f Dissociation half-life.

g Concentration of half-maximum binding.

To confirm target cell specificity, different cancer cell lines expressing varying levels of EGFR were incubated with fluorescein-conjugated repebodies and visualized using confocal laser scanning microscopy (CLSM). The dye-to-repebody labeling ratio was adjusted close to 2 to improve the experimental reproducibility. The emitted fluorescence signals of conjugated repebodies were found to be nearly identical to each other (Figure S3). Consistent with the ELISA results, confocal images showed that the repebody rEgH9 can specifically recognize target cancer cells in an EGFR-dependent manner (Figure 2D) and revealed that the repebody with a smaller *K*_D_ value has a higher binding ability to target antigens displayed on the tumor cell surface (Figure S5). Thus, the binding affinity and kinetics of EGFR-specific repebodies positively correlated with the *in vitro* tumor-targeting activity (Figure 1).

### Preparation of conjugated repebodies with near-infrared fluorophores

**Figure 3:**
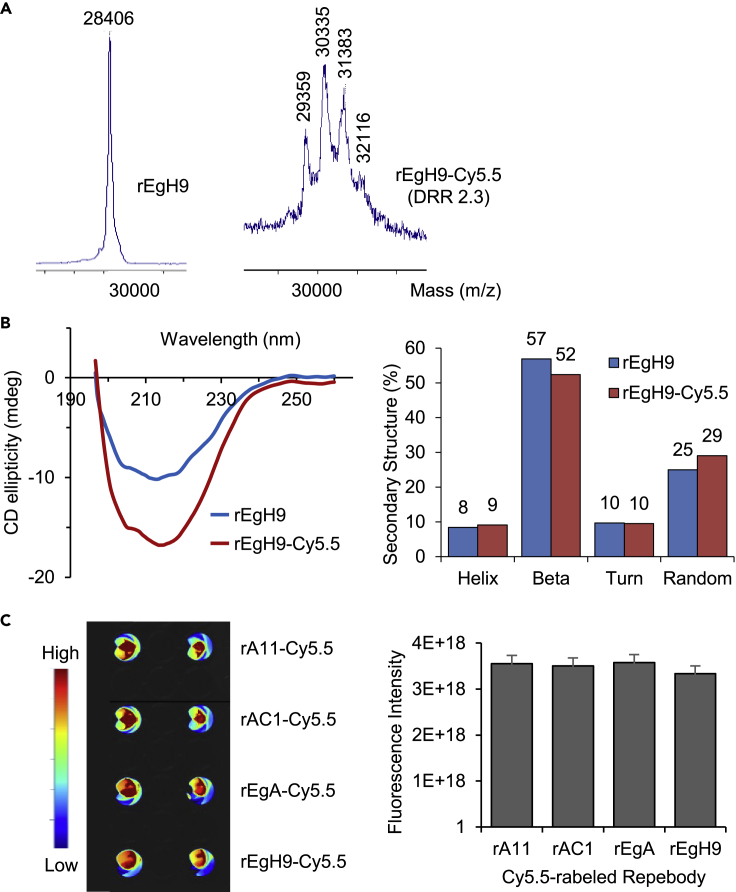
Generation and evaluation of Cy5.5 fluorescence dye-labeled repebodies (A) MALDI-TOF analysis of naked repebodies and rEgH9-Cy5.5 conjugates. The mass peak of rEgH9 significantly shifted upon conjugation by multiples of approximately 1,000, which corresponded to the molecular weight of Cy5.5 dye. The mass spectrum displayed that conjugated repebodies were effectively synthesized with a dye distribution ranging from 1 to 4. (B) Circular dichroism (CD) analysis for the comparison of overall structures of intact and conjugated repebodies. CD spectra (left) and secondary structure analysis (right) revealed that dye conjugation has a negligible effect on secondary structures of dye-labeled repebodies. (C) Fluorescence imaging of the repebody-Cy5.5 conjugates in a solution with an equivalent dye-to-protein molar ratio and concentration using the VISQUE bio-imaging system (left). Quantification of fluorescence signal showed that all the four conjugated repebodies have similar fluorescence intensities (right). The error bars indicate standard deviations of triplicate experiments.

To monitor *in vivo* tumor targeting and localization, the repebodies were coupled to near-infrared (NIR) cyanine 5.5 (Cy5.5) dyes, which are suitable for deep-tissue optical imaging with low background autofluorescence using the N-hydroxysuccinimide ester reaction with amines. In this process, unlabeled repebodies may compete with dye-labeled ones for tumor localization *in vivo*, producing false-negative results with apparently reduced tumor-binding signals. Therefore, the reaction conditions for naked repebody exclusion were optimized, and the stoichiometry of dyes on the repebody was analyzed using the matrix-assisted laser desorption/ionization time-of-flight (MALDI-TOF) mass spectrometry (Figures 3A and S6). When the dye-to-repebody ratio (DRR) was higher than 2, a negligible peak of intact repebodies (m/z 28,406) was observed in mass spectrometry, indicating that almost all repebodies reacted with the NIR dyes. Based on the results, a DRR of 2–3 was determined to be optimal for generating a repebody-Cy5.5 conjugate (Figure S7).

Subsequently, to identify whether the dye conjugation can cause conformational changes in the repebodies and affect biological functions, circular dichroism (CD) spectroscopy was performed. As shown in Figure 3B, the rEgH9-Cy5.5 conjugates displayed CD spectra patterns similar to those of intact repebodies. Based on the CD spectra, we estimated the secondary structure contents in both naked and conjugated repebodies using the secondary structure estimation software, revealing that Cy5.5-labeled repebodies exhibited a native-like content of secondary structure with a high β-sheet content of up to 50%. Additionally, Octet data and competitive ELISA provided that dye-conjugated repebodies have a similar binding affinity compared with the naked ones (Table 1 and Figure S8). These results indicated that fluorophore conjugation has an insignificant effect on the overall structure and the binding capability of the repebodies. There was also a similar range of fluorescence intensity in the four different repebody-dye conjugates with 2.2 DRR (Figure 3C). Considering the biochemical properties compared with their native forms, conjugated repebodies can thus facilitate the accurate analysis of *in vivo* distribution and tumor localization according to differences in affinity while minimizing unpredictable variables.

### Cell-based analysis of the target-binding ability of repebodies

**Figure 4:**
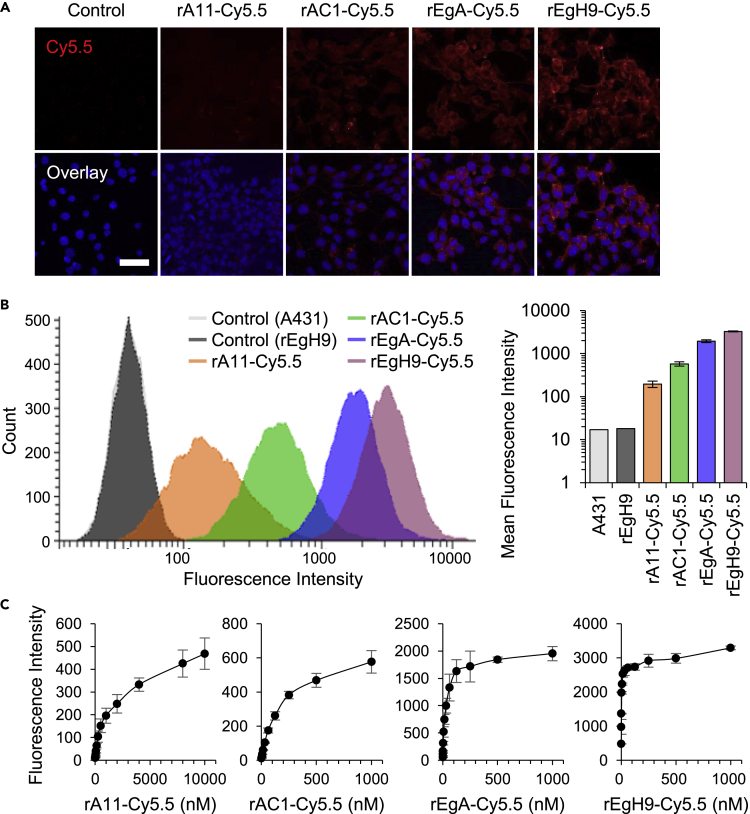
Assessment of target cell-binding ability of Cy5.5-conjugated repebodies (A) Confocal images of Cy5.5-labeled repebodies. Four kinds of conjugated repebodies (10 μg/mL) were incubated with the A431 cells for 3 h, followed by washing and imaging using confocal microscopy. Control indicated the untreated cells. The nuclei are stained with Hoechst 33342 (blue). Scale bar, 50 μm. (B) Fluorescence-activated cell sorting (FACS) analysis of conjugated repebodies. A431 cells were incubated with 100 μg/mL of naked rEgH9 or repebody-Cy5.5 conjugates, and the stained cells were subjected to flow cytometry (left). Control indicated untreated A431 cells. The bar diagram represents mean fluorescent intensities (MFI) of conjugated repebodies for comparing the relative binding ability for target cells (right). (C) Binding curves of repebody-Cy5.5 conjugates to the A431 cells. The MFI values were plotted against varying concentrations of labeled repebodies. Based on the FACS data, half-maximum binding concentrations (*K*_1/2_) of respective repebodies were determined as given in Table 1. The error bars indicate standard deviations of triplicate experiments.

Before the *in vivo* study, the *in vitro* target-binding ability of repebody-Cy5.5 conjugates was investigated using the CLSM and fluorescence-activated cell sorting (FACS) analysis. All the conjugated repebodies were treated with various cancer cells for 3 h, and red fluorescence of cells was visualized by confocal microscopy. As a result, strong fluorescence signals were observed in the EGFR-overexpressing A431 and MDA-MB-468 cells in proportion to the binding affinity of repebodies (Figures 4A and S9). However, no detectable fluorescence was observed in the MCF7 cells expressing low levels of EGFR. FACS analysis was performed to quantify the differences between the four repebodies in binding the target cell. Consistent with the result of the fluorescence imaging, a gradual increase in the median fluorescence intensity was observed in an affinity-dependent manner with a distinguishable shift in emission peaks compared with both non-treated and unlabeled repebodies-treated A431 cells (Figure 4B). The results indicated that an increased probability of binding to the target cells *in vitro* is attributed to the higher binding affinities of repebodies, as illustrated in Figure 1. Furthermore, the dose-response binding curves of the repebody-Cy5.5 conjugates to A431 cells were determined to calculate the apparent functional affinities (Figure 4C). It revealed that the calculated half-maximum binding (*K*_1/2_) values are in good agreement with the equilibrium binding constants (*K*_D_) estimated from the Octet analysis (Table 1). The *K*_1/2_ values larger than *K*_D_ might be a consequence of the equilibrium shift resulting from relatively long and repetitive washing steps in flow cytometry, implying that the antigen-binding behavior of repebodies can be significantly affected by the surrounding environment where the antigen is located.

### Investigation of *in vivo* tumor localization of repebodies

**Figure 5:**
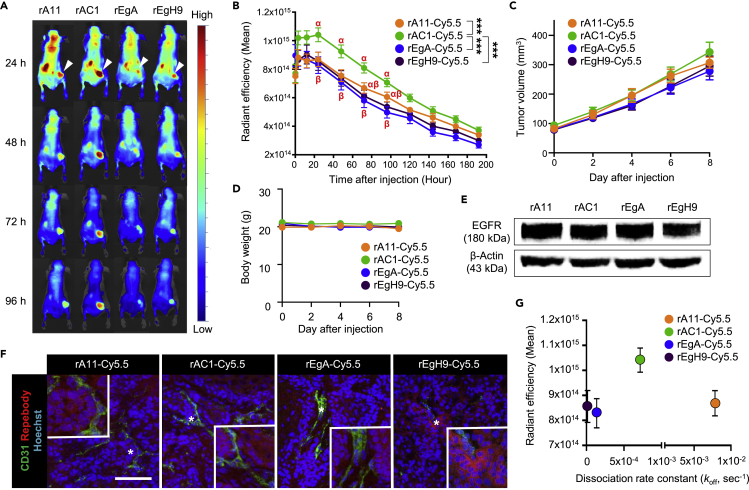
*In vivo* analysis of tumor targeting and localization of Cy5.5-conjugated repebodies (A and B) (A) Representative whole-body biodistribution images and (B) the mean radiant intensities measured in the tumor at different time points after intravenous injection of all repebody-Cy5.5 conjugates were administered in the A431 tumor-bearing mice. A clear increase in the accumulation of rAC1-Cy5.5 conjugates was observed after 6 h. White arrowheads in the images at 24 h indicate the location of the subcutaneous tumor. ∗∗∗ indicates p < 0.001, calculated by one-way ANOVA. Data are the mean ± SEM (n = 10 mice per rAC1-Cy5.5 conjugate-treated group and n = 11 mice per the other conjugate-treated groups) and mean values with the different alphabet (α, β) represent the different pairs of values (p < 0.05). (C) Growth of the A431 tumor in mice injected with the conjugated repebodies having different binding affinity. There was a negligible difference in tumor volume among the four Cy5.5-labeled repebodies. Data represent the mean ± SEM. (D) Kinetics of body weight in tumor-bearing mice treated with repebodies. Data are the mean ± SEM. (E) Western blot for the *ex vivo* A431 tumor from the repebody-treated groups for EGFR. β-Actin was used as the loading control. (F) Immunostaining of repebody (red) and CD31 (green) in the A431 tumors grown in mice. CD31 is used as a vascular marker. Nuclei are shown in blue with Hoechst 33342 counterstaining. Compared with the other three repebody-Cy5.5 conjugates that penetrated and distributed through the entire tumor tissues at 6 h after injection, rEgH9-Cy5.5 conjugates showed a restricted localization to the perivascular space. The inset shows magnified regions indicated with an asterisk (∗). Scale bar, 100 μm. (G) The mean radiation efficiency (20 h after injection) of Cy5.5-conjugated repebodies were plotted with respect to the dissociation rate constants (*k*_off_) for EGFR. Data represent the mean ± SEM.

To study the affinity dependence of repebodies on tumor localization, the EGFR-overexpressing A431 cells were subcutaneously implanted into athymic nude mice and the repebody-Cy5.5 conjugates were injected intravenously to track the whole-body kinetics. The optimal dose of conjugated repebodies for molecular imaging with good tumor-to-normal tissue contrast was established by treating various concentrations of rEgH9-Cy5.5 (1, 3, and 7.5 mg/kg) into A431 tumor-bearing mice (Figure S10). As a result, EGFR-specific repebodies exhibited efficient tumor accumulation in a dose-dependent manner and preferential renal clearance, as the primary excretion route for other small-sized proteins (Zahnd et al., 2010; Vazquez-Lombardi et al., 2015). Based on the preliminary data, 5 mg/kg (a median value between 3 and 7.5 mg/kg) was selected as the proper dose for monitoring tumor localization in the xenografts, and an *in vivo* study was conducted using all four different conjugated repebodies. It was revealed that all repebodies rapidly accumulated in the tumor upon administration, and the fluorescence of tumor-localized repebodies was long retained until at least day 8, whereas they were quickly eliminated from normal tissues (Figures 5A and 5B). Interestingly, we found a tendency that the tumor localization is reduced as the affinity increases above a certain threshold, as previously reported (Adams et al., 2001; Saga et al., 1995; Tsumura et al., 2018). It is noteworthy that the rAC1-Cy5.5 conjugates with a moderate affinity (*K*_D_) of 3.5 nM exhibited the highest localization and retention in tumor tissue until day 4, even though the radiant efficiency of untreated tumors was almost comparable among all the groups. No signs of tumor growth retardation or systemic toxicity were observed in the repebody-treated xenografts (Figures 5C and 5D). These results suggest that the *in vivo* tumor localization is not directly proportional to binding affinity. Thus, finding the optimal affinity can lead to an improved tumor localization of therapeutic proteins.

Previous studies in support of the binding-site barrier effect have reported that high-affinity antibodies tend to accumulate around the blood vessels surrounding the tumor, which is responsible for restricting the deep penetration into the tumor (Saga et al., 1995). To identify the localization pattern of repebodies in tumor mass, *ex vivo* western blot analysis and immunofluorescence studies were performed in the repebody-Cy5.5 conjugate-treated groups of the A431 tumor xenografts. As evident in Figure 5E, all the tumor tissues had the same level of EGFR expression. We found that the Cy5.5-conjugated rEgH9 having the highest affinity (*K*_D_) of 51 pM dominantly localized around CD31-positive tumor vascular cells, whereas the relatively low-affinity binders, including rA11, rAC1, and rEgA, evenly distributed throughout the tumor (Figure 5F). As previous studies involving fragmented or conjugated antibodies (Adams et al., 2001; Tsumura et al., 2018), repebodies clearly demonstrated that the high affinity with slow dissociation rates restricted tumor penetration and localization *in vivo* (Figure 5G). Considering that the extent of drug delivery into tissues is typically reflected as a therapeutic activity, our findings suggest that systemic study of the effects of binding kinetics and affinity on tumor localization holds great promise for potentiating the anti-cancer activity of protein drugs by overcoming the binding-site barrier effect.

## Discussion

In the development of therapeutic proteins and antibodies, binding affinity is considerably one of the most crucial factors to achieve remarkable on-target activities in terms of high specific targeting and preferential localization in the diseased tissues (Thurber et al., 2008a, 2008b; Carter, 2001). Thus, attempts have been made to improve the binding affinity for developing highly potent neutralizing antibodies through affinity maturation, although it is a time-consuming and laborious process (Tabasinezhad et al., 2019). However, there is still no clear criteria regarding the optimal affinity for the greatest *in vivo* efficacy, which increases the dilemma of whether to proceed with affinity maturation. Furthermore, several studies have suggested the binding-site barrier effect where a certain level of binding affinity can elicit higher *in vivo* tumor localization of antibodies compared with the highest affinity binder (Rudnick et al., 2011; Adams et al., 2001; Tsumura et al., 2018). Unfortunately, there is no substantial evidence to counter the controversy over the affinity-dependent increase in tumor localization *in vivo* due to limitations in securing a group of binding proteins having a wide range of affinities and sharing identical biochemical properties. To address this issue, the impact of binding affinity on *in vivo* tumor localization of repebodies (a small-sized protein binder) was evaluated. Four different repebodies with a wide range of EGFR-binding affinities spanning three orders of magnitude have been previously generated based on a stepwise modular evolution (Lee et al., 2015). Given the significant level of amino acid sequence identity (>95%), the group of EGFR-specific repebodies can be considered as an ideal starting point to clarify whether tumor localization of binding proteins is proportional to their target-binding affinities. Moreover, the remarkable biochemical stability of repebodies contributes to the facile synthesis of fluorescent dye-conjugates with negligible aggregation and uniform stoichiometry, allowing accurate analysis of *in vivo* distribution and accumulation.

As illustrated in Figure 1A because most of the *in vitro* binding experiments are conducted in a closed system, all protein binders generally display a traditional Langmuir binding behavior at equilibrium (Hulme and Trevethick, 2010). Consistent with the Langmuir isotherm model, *in vitro* cell-based assays, including fluorescence imaging and flow cytometry, exhibited the affinity-correlated binding signals for conjugated repebodies in cancer cells. However, a proportional relationship between binding affinity and tumor localization was not observed when repebody-dye conjugates were administered to the EGFR-overexpressing tumor xenografts. Instead, rAC1 with a moderate affinity (*K*_D_ of 4 nM) demonstrated a faster rate of tumor accumulation than the other high-affinity binders, rEgA and rEgH9. Moreover, the highest affinity repebody rEgH9 (*K*_D_ of 51 pM) preferentially localized in perivascular regions of tumors, implying that an affinity exceeding a certain threshold can lead to limited penetration of repebodies in tumor tissues. Unlike *in vitro* (closed) systems, the tumor microenvironment *in vivo* is typically considered an open system in which continuous exchange of biological materials is allowed (Gabrielsson et al., 2018; Copeland, 2016). Thus, it can be speculated that when repebodies are actively transported from the systemic circulation into the tumor mass, their binding kinetics to tumor-surface antigens can be dynamically controlled by several physiological factors such as diffusion and interstitial transport as well as lymphatic drainage (Figure 1B), leading to differences in tumor-targeting ability and penetration capability *in vitro* and *in vivo*.

Taking into account the *in vivo* transport of repebodies into the tumor under steady-state conditions, the binding kinetic parameters such as rate constants of association (*k*_on_) and dissociation (*k*_off_) were postulated to be more suitable for dissecting the factors affecting the tumor penetration and localization of repebodies than the equilibrium dissociation constant, denoted as *K*_D_. The Octet binding data showed sequentially decreased dissociation constants by approximately one order of magnitude for each level from the lowest affinity rA11 (*K*_D_ of 14 nM) to the highest affinity rEgH9, as presented in Table 1. It is noteworthy that three repebodies, including rAC1, rEgA, and rEgH9, have different *K*_D_ values derived from changes in the dissociation rate constants (*k*_off_) for EGFR, not the association ones (*k*_on_). Moreover, the calculated dissociation half-lives (*t*_1/2_) of rEgA and rEgH9 are 84 and 775 min, respectively, which are much longer than that of rAC1, 16 min. The off-rate, a concentration-independent parameter, can be considered an important factor driving the therapeutic proteins to penetrate deep inside the tumor mass through interstitial diffusion. This is because only proteins (or ligands) in the unbound state can be freely transported within the interstitial space (Thurber et al., 2008a, 2008b). Thus, it can be implied that the protein binders with a relatively short dissociation half-life are more advantageous for accumulation into the tumor compared with the tight binders that do not dissociate well from the target receptors. Based on the binding kinetics data and *in vivo* results, it can be concluded that repebody rAC1 localizes within an optimal range of dissociation rate constant and dissociation half-life, leading to the greatest tumor accumulation in a short time through a highly balanced reversible binding and efficient interstitial transport. On the other hand, the higher-affinity binders, rEgA, and rEgH9, exhibiting relatively low off-rates and almost the same on-rates as rAC1 might be preferentially deployed around the tumor vascular niche to form stable repebody-EGFR binary complexes, resulting in poor interstitial transport and limited tumor localization. As shown in Figure 5F, the expected *in vivo* binding-site barrier was demonstrated through multiplexed tissue imaging of rEgH9-treated tumor xenografts. Considering that the dissociation half-life of rEgA is 84 min, it is feasible that their binding signals around tumor blood vessels were not insignificant after 6 h (360 min) of incubation, which is sufficient time to allow repetitive reversible binding reactions and interstitial diffusion. This result implies that the binding-site barrier can be considered as a kinetically controllable *in vivo* phenomenon (Singh et al., 2020; Vauquelin, 2016).

The existence of the optimal affinity and binding-site barrier effect discovered in the *in vivo* study of repebody is consistent with previous results of single-chain variable fragments (scFvs) and antibodies (Rudnick et al., 2011; Adams et al., 2001; Saga et al., 1995; Tsumura et al., 2018), but discordant with the case of very small proteins, DARPins (Zahnd et al., 2010). Tumor accumulation of proteins can be largely affected by their molecular weight as well as binding affinity. In previous reports, small-sized proteins (∼15 kDa) were believed to have substantially higher vascular permeability and better tumor interstitial diffusivity compared with protein binders with intermediate molecular size (∼25 kDa), which can result in size dependency on tumor localization as computationally predicted (Debie et al., 2020; Zahnd et al., 2010; Schmidt and Wittrup, 2009). Therefore, the limited extravasation and hindered interstitial diffusion due to relatively large molecular weight could affect the different aspects of tumor localization including binding-site barrier effect. Along with molecular size, the dosage of protein binders can also play an important role in tumor localization and therapeutic outcome (Singh et al., 2020). Similar to the relationship between binding affinity and the binding-site barrier effect, increasing the administered dose is associated with the stable formation of antigen-binding protein binary complexes in tumors, strengthening the binding-site barrier effect. Interestingly, a previous study displayed that a considerable reduction in tumor localization of the highest affinity scFvs (*K*_D_ = 15 pM for HER2) was more clearly observed in anephric mice than normal mice (Adams et al., 2001). Considering that the anephric mice are not capable of fast renal clearance, it is reasonable to deduce that the restricted tumor uptake is the consequence of a prolonged and elevated level of the scFv in the blood. On the other hand, it is anticipated that the binding-site barrier effect could be overcome if the administered dose far exceeds the concentration at which saturation binding between tumor antigens and injected proteins can be achieved. In this situation, plasma concentration of protein binders is sufficiently maintained at high levels, leading to diminishing free tumor antigens near blood vessels and weakening the binding-site barrier effect. As a result, passively transported protein binders to tumors by continuous extravasation can pass through the vascular surrounding tissue without being trapped by surface antigens, allowing deeper interstitial diffusion and tumor penetration. From a similar perspective, it was reported that the binding-site barrier effect can be effectively attenuated in tumors expressing lower levels of antigens at the same dose, because the amount of free antigens is more dramatically decreased than in antigen-overexpressed tumors (Singh et al., 2020). Collectively, the relationship between tumor localization and various biochemical properties of proteins still remains a matter of scientific debate. Further studies should thus be conducted with a systematic evaluation of uncertain *in vivo* factors to clarify the aforementioned issues.

In summary, the present study demonstrates the binding-site barrier effect using EGFR-specific repebodies. Affinity maturation is an inevitable process for improving both the target specificities and therapeutic potencies of binding proteins during the lead optimization phase. Despite technical advances in molecular and computational biology, this process is still regarded as time-consuming and labor-intensive (Tabasinezhad et al., 2019). Moreover, given that a moderate binding affinity may result in the highest tumor penetration and accumulation rather than higher affinities, it can be expected that a study similar to a dose-ranging trial, exploring affinities, can be as effective as affinity maturation to improve therapeutic benefits. Taken together, understanding the influence of binding kinetics and affinity on *in vivo* behavior can practically guide in streamlining the discovery and optimization of protein therapeutics.

### Limitations of the study

In this study, we evaluated the correlation between binding affinity and tumor localization of protein binders, called repebody, through near-infrared fluorescence molecular imaging in EGFR-overexpressing tumor xenografts. We obtained statistically significant results that can prove that intermediate affinity binder has better tumor localization than very-high-affinity ones. However, it may be premature to expect that the increased tumor localization translates directly into the enhancement of therapeutic benefits. Therefore, further studies using drug-conjugated repebodies are needed to validate the effectiveness of affinity-based protein design and development for cancer treatment. As reported in previous studies, various biochemical properties of protein binders including molecular weight, valency, and binding mode have been well known to substantially affect their pharmacological effects, especially tumor targeting and localization. Considering that difference in the molecular size of proteins can lead to significant changes in the rates of extravasation, interstitial diffusion, and systemic clearance, this study focused primarily on binding affinity, and the binding-site barrier effect may have limitations in general application to some proteins with unique characteristics. To expand our findings, it is inevitable to systematically evaluate the various influencing factors for tumor localization of proteins based on size, avidity, and binding epitope.

### Resource availability

#### Lead contact

Further information and requests should be directed to and will be fulfilled by the Lead Contact, Joong-jae Lee (leejj@kangwon.ac.kr).

#### Material availability

This study did not generate new materials.

#### Data and code availability

All the data are available within the article.

## Methods

All methods can be found in the accompanying Transparent methods supplemental file.

## Supplemental information

Document S1. Transparent methods and Figures S1–S10
