# Supplementary material for: Dissecting the impact of target-binding kinetics of protein binders on tumor localization
Source: iScience. 2021 Jan 29;24(2):102104. doi: 10.1016/j.isci.2021.102104 (PMC7881221; doi:10.1016/j.isci.2021.102104)
Supplement: Document S1. Transparent methods and Figures S1–S10 [file mmc1.pdf]

iScience, Volume *24*

Supplemental Information

Dissecting the impact of target-binding kinetics of protein binders on tumor localization

Yunjin Song, Hoibin Jeong, Song-Rae Kim, Yiseul Ryu, Jonghwi Baek, Jinhak Kwon, Hyeongjun Cho, Kil-Nam Kim, and Joong-jae Lee

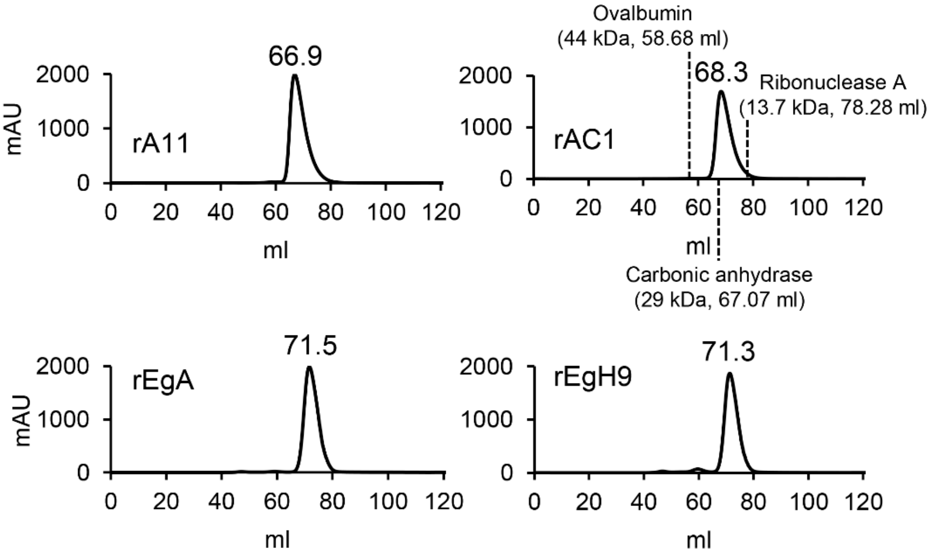

Figure S1. Gel Permeable Chromatography (GPC) profile of the EGFR-specific repebodies (rA11, rAC1, rEgA and rEgH9), Related to Figure 2. Four repebodies were subjected to GPC using HiLoad 16/60 superdex 75 column after Ni-NTA purification. All of the monomeric repebodies (28 kDa) were eluted as a single major peak around 70 mL of elution volume. For comparison, respective elution peaks of ovalbumin, carbonic anhydrase, and ribonuclease A were marked with their respective molecular weight.

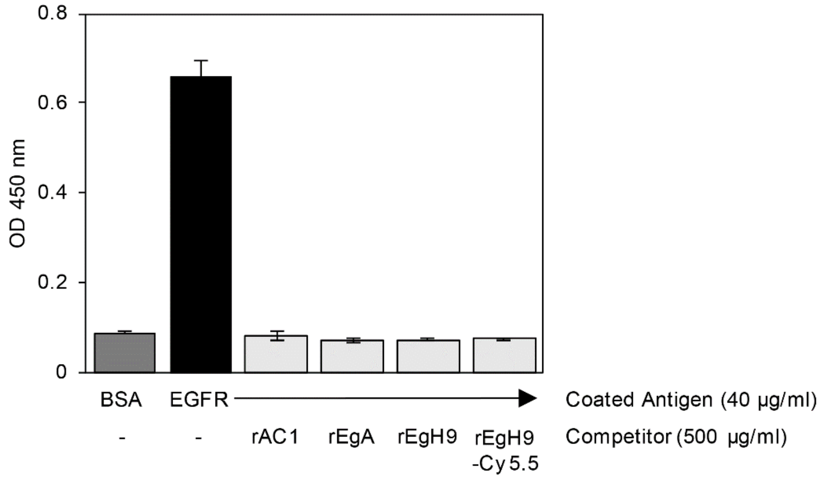

Figure S2. Competitive ELISA for identifying a binding mode of all EGFR-specific repebodies, Related to Figure 2. All antigens were coated at 40 µg/ml, and BSA was used as a negative control. Repebody rA11 was biotinylated to generate binding signals for EGFR by using HRP-conjugated streptavidin. Excess soluble competitors (500 µg/ml) were co-incubated with biotinylated rA11, and the resulting mixture was introduced to each antigen coated well. As results, three types of affinity-maturated repebodies (rAC1, rEgA and rEgH9) and dye-conjugated rEgH9 significantly decreased the binding signals of rA11, the initially selected binder for EGFR. Given that the large molecular weight (~110 kDa) of the EGFR ectodomain composed of 4 discrete domains, the ELISA data presented that all repebodies used in this study bind a common epitope on EGFR. The error bars indicate standard deviations of triplicate experiments.

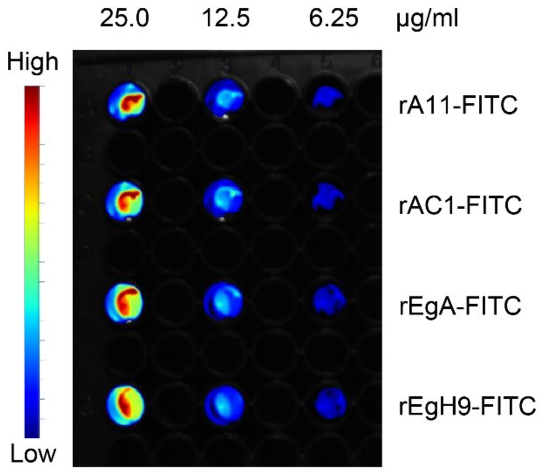

Sample	Reaction Ratio (FITC to Repebody)	Conjugation Raito (FITC to Repebody)
rA11-FITC	12 : 1	2.17
rAC1-FITC	11 : 1	2.18
rEgA-FITC	13 : 1	2.13
rEgH9-FITC	12.5 : 1	2.17

Figure S3. Conjugation of EGFR-specific repebodies with equivalent amount of fluorescein dye, Related to Figure 2. All repebodies were conjugated with NHS-fluorescein under optimized conditions, and resulting FITC-conjugated repebodies showed the dye-to-protein ratio close to 2 (lower). Fluorescence intensities of the dye-conjugated repebodies were visualized using a ViSQUE bio-imaging system (upper). The fluorescence image indicated that all four conjugated repebodies have the similar fluorescence intensity at each concentration.

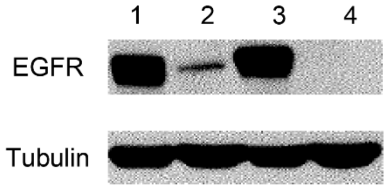

	Cell Line	Origin	Expression Level
1	A431	Squamous Carcinoma	+++
2	HCC827	Lung Adenocarcinoma	+
3	MDA-MB-468	Breast Adenocarcinoma	+++
4	MCF7	Breast Adenocarcinoma	-

Figure S4. Western blot analysis of various tumor cells to assess the level of expression of EGFR, Related to Figure 2. MCF7 cells were used as a control for very low EGFR expression. The other three cell lines (A431, HCC827 and MDA-MB-468) were observed to overexpress EGFR. Tubulin (52 kDa) was used as the loading control.

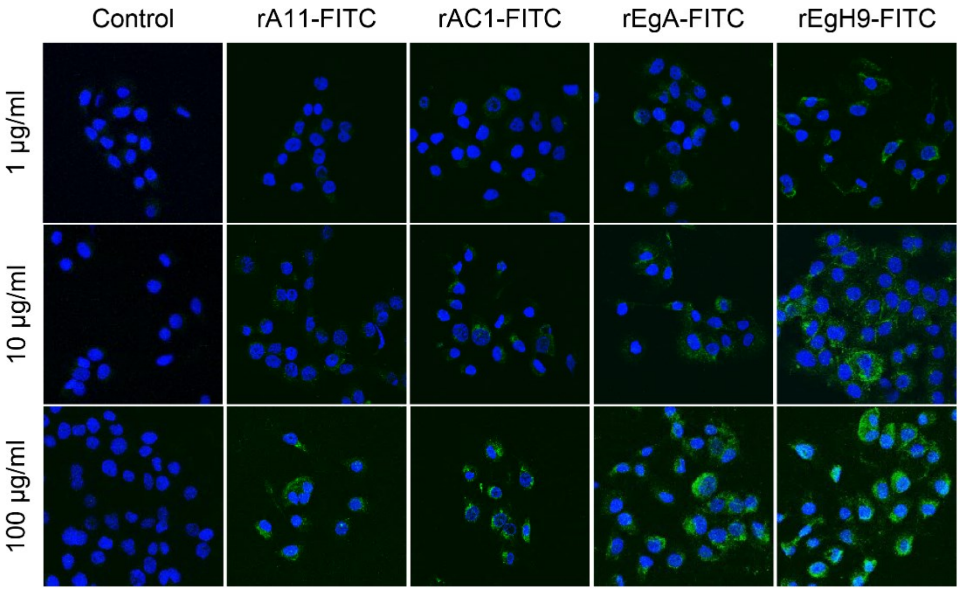

Figure S5. Confocal images of A431 cells treated with dye-labeled repebodies, Related to Figure 2. Four kinds of conjugated repebodies with various concentration of 1, 10 and 100 µg/ml were incubated with A431 cells for 3 hours, followed by washing and imaging using a confocal microscope. The nuclei are stained with Hoechst 33342 (blue).

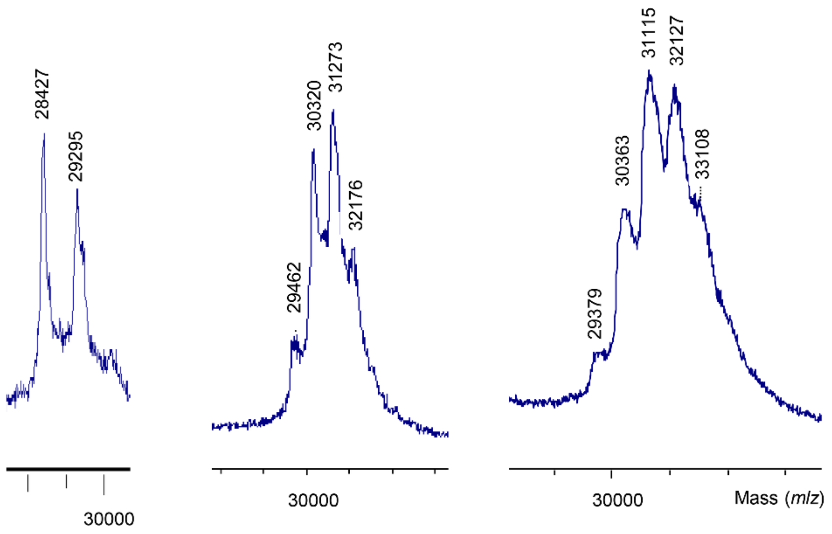

rEgH9-Cy5.5			
Reaction Ratio (Dye : Repebody)	8:1	14:1	20:1
Dye to Repebody Ratio (DRR)	1.29	3.39	3.30

Figure S6. Characterization of dye-conjugated repebodies using mass spectrometry, Related to Figure 3. MALDI-TOF mass spectra of rEgH9-Cy5.5 conjugates showing various dye-to-repebody ratios (DRR) when the reaction ratios are 8:1 (left), 14:1 (middle), and 20:1 (right). While the peak indicating naked rEgH9 (m/z 28,427) was still observed in DRR of 1.29, it was completely not detected in the other two rEgH9 conjugates having multiple peaks of dye-conjugated form and DRR over 3.

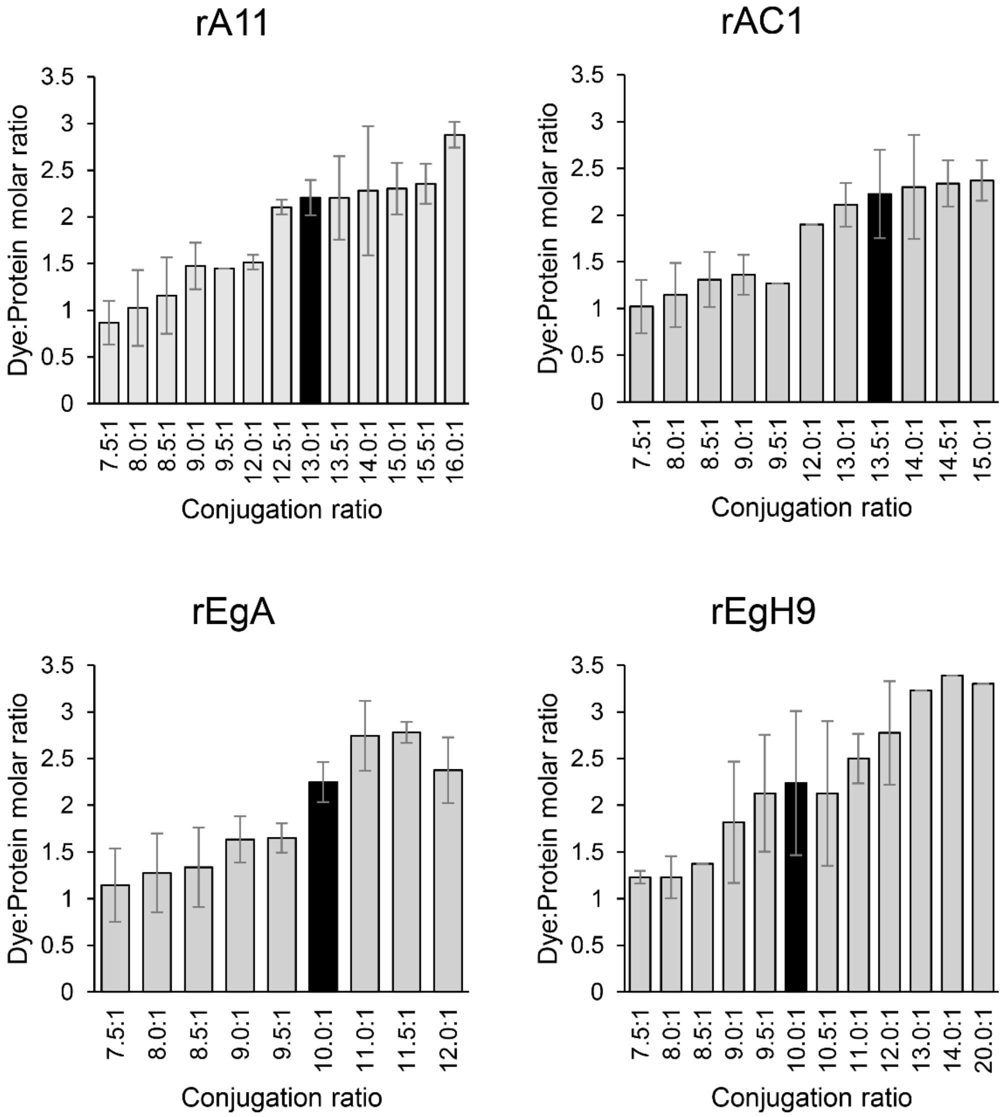

Figure S7. Establishment of reaction conditions for generating dye-conjugated repebodies, Related to Figure 3. Conjugation of repebody with Cy5.5 dye using various reaction ratio for determining the conjugation ratio that results out dye-to-repebody ratio (DRR) of about 2 (represented as a black bar in each repebody). Generally, as the molar ratio of Cy5.5 dyes to repebodies was increased, DRR tended to increase. Based on these results, we optimized the conjugation conditions for all four repebodies. The error bars indicate standard deviations of multiple experiments.

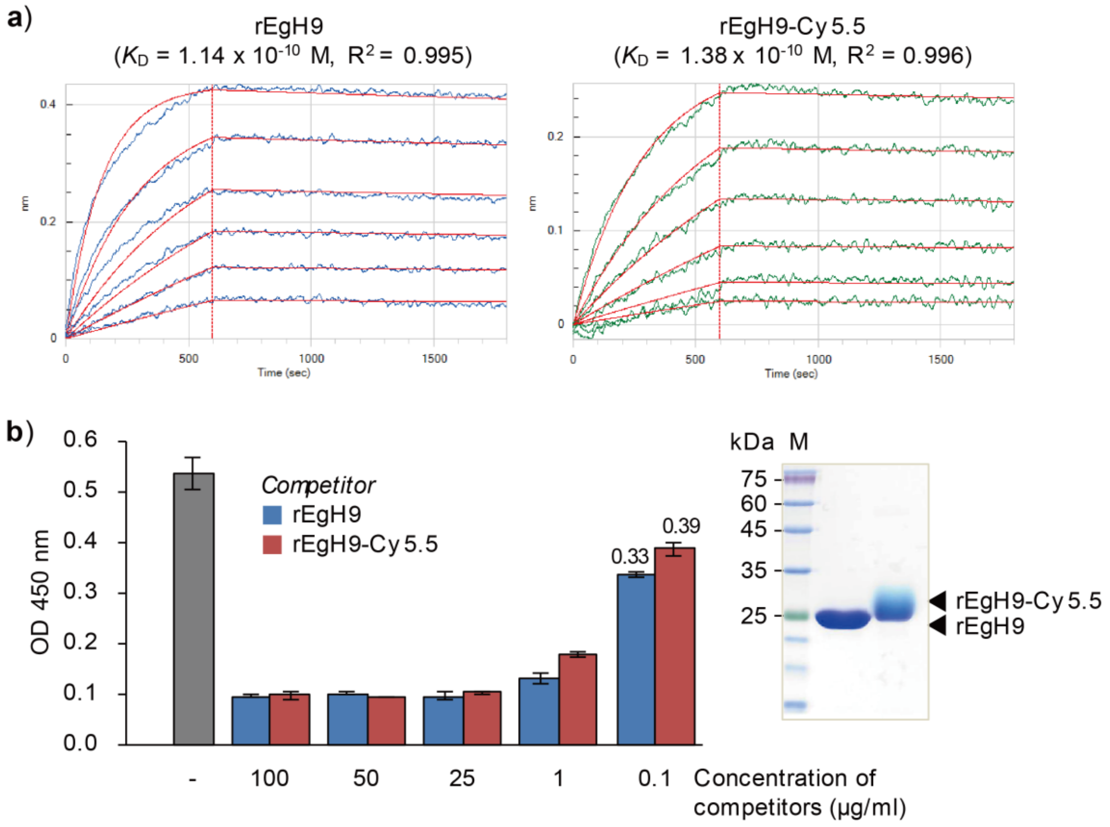

Figure S8. Octet and competitive ELISA data of intact and dye-conjugated repebody rEgH9, Related to Figure 3. (**a**) Octet analysis was performed to evaluate the effect of dye conjugation on the binding affinity of repebodies. As results, Cy5.5-conjugated repebodies showed a similar dissociation constant (K_D) of 138 pM as naked repebodies (K_D = 114 pM), proving that conjugated dyes on repebodies have a negligiable effect on the binding ability. We observed a slight difference in the measured K_D values of rEgH9 between this experiment and **Table 1** (K_D = 51 pM), which could be considered an acceptable level of batch-to-batch variations. (**b**) The competitive assay was likewise conducted as described in **Figure S2**. ELISA data showed that the conjugated repebodies inhibit the binding of biotinylated rA11 comparable to intact repebodies, indicating that these two types of repebodies (rEgH9 and rEgH9-Cy5.5) have a similar level of binding affinity for EGFR. The error bars indicate standard deviations of triplicate experiments. SDS-PAGE displayed bands of intact and conjugated repebodies (1 mg/ml) with relative band intensities calculated as 1.0 and 0.8, respectively.

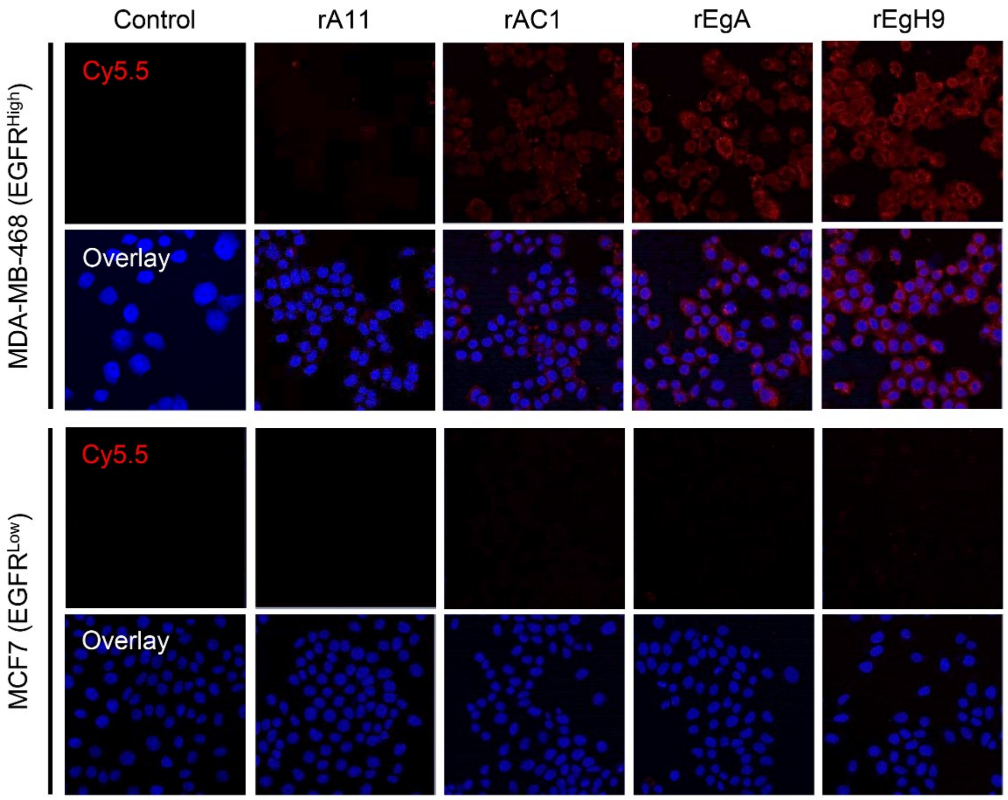

Figure S9. Confocal images of MDA-MB-468 and MCF cells treated with Cy5.5-labeled repebodies, Related to Figure 4. Cy5.5-repebody conjugates (10 µg/ml) were incubated with the cells expressing different levels of EGFR for 3 hours, and cells were washed and fixed, followed by imaging using confocal microscope. The nuclei are stained with Hoechst 33342 (blue).

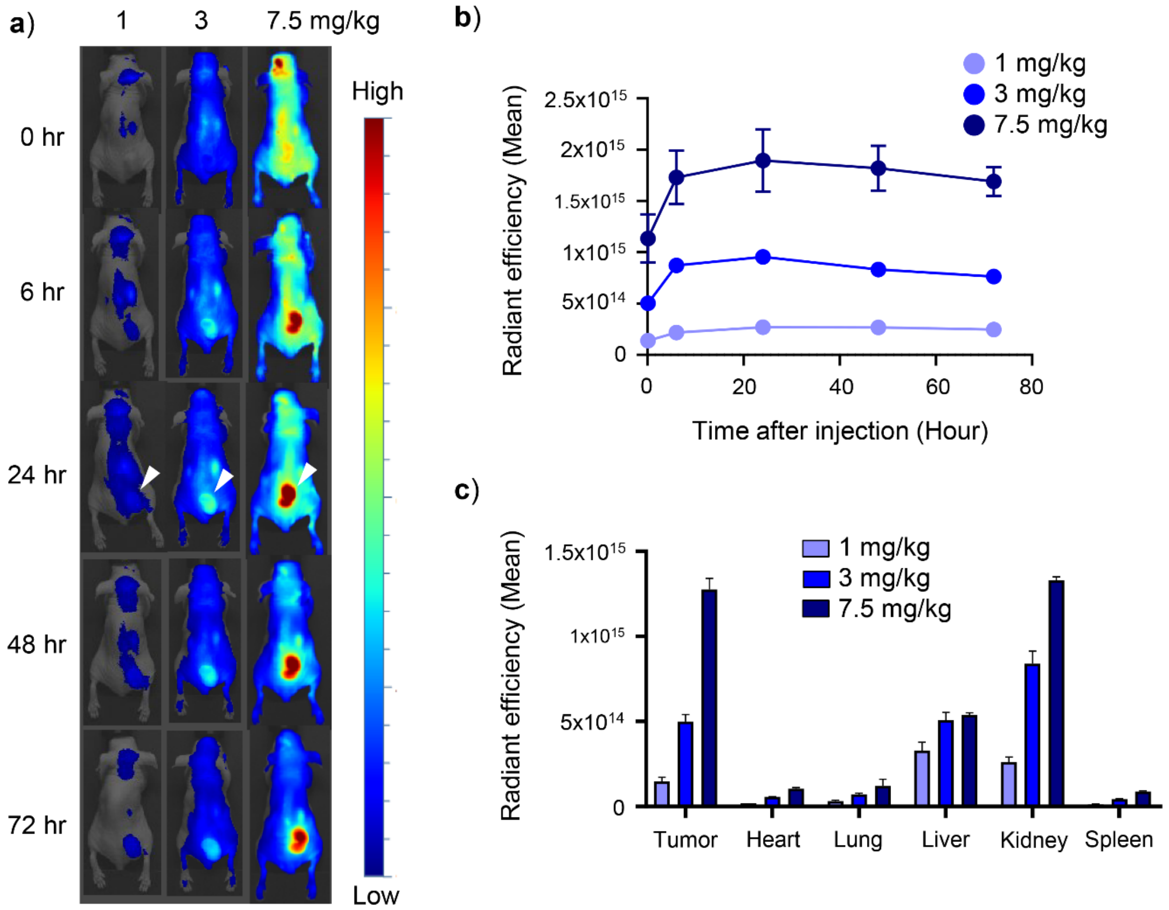

Figure S10. Preliminary *in vivo* studies to determine the optimal injection dose of repebody-Cy5.5 conjugates for molecular imaging, Related to Figure 5. (**a**) Whole body bio-distribution profiles demonstrating specifically tumor targeting of rEgH9-Cy5.5 with a concentration of 1, 3, and 7.5 mg/kg in A431 subcutaneous tumor model. (**b**) Quantification of rEgH9-Cy5.5 accumulated in tumors as presented in **panel (a)**. Data are the mean ± SEM for two mice per group. (**c**) Mean radiant efficiency in A431 tumor or organs harvested at 72 hr after treatment of rEgH9-Cy5.5. Data are the mean ± SEM for two mice per group.

Transparent Methods

Expression and purification of repebodies

Four classes of repebodies (rA11, rAC1, rEgA, and rEgH9) were cloned into pET21a vector (Novagen, USA) using NdeI and XhoI restriction sites. The resulting recombinant vectors were transformed into Origami B (DE3) host cells (Novagen, USA). After transformation, single colony was inoculated in Luria-Bertani (LB, Duchefa, Netherlands) medium containing 100 μg/ml ampicillin and cultured at 37°C overnight. Next day, overnight culture was diluted 1:100 into fresh LB media and grown until OD600 reached about 0.5. Repebody expression was induced by addition of 0.5 mM of IPTG and further incubated at 18 °C for overnight. Cell harvest was conducted by using centrifugation at 4,000 rpm for 30 min. After resuspending cell pellet with lysis buffer (20 mM Tris (pH 8.0), 300 mM NaCl, and 10 mM Imidazole), repebody purification was performed as described elsewhere (Lee et al., 2012). Briefly, soluble fraction of disrupted cells after centrifugation at 13,000 rpm for 60 min was filtered using 0.22 micron syringe filters. Repebodies were isolated using Ni-NTA agarose (Qiagen, USA) and further purified on a HiLoad 16/60 superdex 75 pg (GE Healthcare, USA). The resulting repebodies were eluted with PBS (pH 7.4). Purity and concentration of repebodies were checked using SDS-PAGE and UV-spectroscopy.

Enzyme-linked immunosorbent assay

For the target specificity of repebodies, 10 μg/ml of soluble proteins (BSA, human EGFR, Trypsin, mOrange, and IL-6) were coated onto 96 well MaxiSorp plate (SPL, Korea) at 4 °C for overnight. After 3 times of PBS (pH 7.4) washing, each well was blocked with blocking buffer (PBS supplemented with 0.1% Tween-20 and 2% BSA) at room temperature for 1 hour. Biotinylated repebodies (10 μg/ml) were diluted with blocking buffer and added into each well for 1 hour. After 3 times of PBST (PBS supplemented with 0.1% Tween-20) washing, HRP-conjugated streptavidin (1:5000; Bio-rad, USA) was used for the detection of biotinylated repebodies at room temperature for 1 hour. After incubation of HRP-streptavidin conjugates, plate washing was carried out 2 times with PBST and 1 time with PBS. TMB solution was added to amplify the binding signal and 1 N H_2SO_4 was added to stop the reaction. The absorbance was measured at 450 nm using a microplate reader (Molecular Devices, USA). For competitive binding assay, each well was coated with human EGFR (40 μg/ml). Biotinylated repebody rAll (100 μg/ml) was diluted into the blocking buffer with competitors (rACl, rEgA, rEgH9, and rEgH9-Cy5.5; a final concentration of 500 μg/ml).

Affinity measurements using Octet QK384

The repebodies were diluted to 10 μg/ml (rA11, rAC1) and 20 μg/ml (rEgA, rEgH9) using 10 mM Acetate buffer (pH 5). All repebodies were coupled to amine-reactive second-generation (AR2G) biosensors (ForteBio) using 400 mM EDC (1-Ethyl-3-[3-dimethylaminopropyl]-carbodiimide hydrochloride) and 200 mM S-NHS (N-hydroxysulfosuccinimide) with instructions from ForteBio. The human EGFR (Sino Biological, China) was serially diluted two-fold with a concentration range from 100 nM to 0.7813 nM in 1x kinetics buffer. The protein solutions (200 μl) were added into a 96-well black polypropylene microplate (Greiner Bio-One, Kremsmünster Österreich). The measurements were carried out by the Octet QK384 systems (ForteBio, USA). Briefly, the repebodies-loaded AR2G sensors were dipped into wells containing human EGFR to monitor repebody association, followed by a 20-min dissociation phase. All solutions used in the measurements were purchased from ForteBio. The dissociation half-life ($t_{1/2}$), the time that it takes for half of the complex to dissociate was determined from the dissociation rate constant (k_{off}) using the equation $t_{1/2} = \ln 2 / k_{off}$.

Fluorescence dye labeling

The NHS-Fluorescein (Thermo scientific, USA) and Sulfo-Cyanine5.5 (Cy5.5) nhs ester (Lumiprobe, USA) were used as fluorescent dyes for conjugation. In order to conjugate the fluorescent dyes to repebodies, all of the repebodies were dissolved in PBS (pH 7.4) at a final concentration of 1 mg/ml. The dye fluorescein was dissolved in dimethyl sulfoxide (DMSO) to a final concentration of 10 mg/ml. All proteins were labeled with NHS-fluorescein at a dye to protein ratio to be 11 to 13, followed by incubation with continuous stirring at 4°C for 2 hours in the dark. The Cy5.5 dye was dissolved in PBS to a final concentration of 10 mg/ml. All proteins were incubated with Sulfo-Cy5.5 nhs ester at a dye to protein ratio to be 8 to 20, followed by incubation at room temperature for 1 hours in the dark. After centrifugation of the reaction solution at 13,000 rpm for 10 min at 4°C, supernatant was further filtered using 0.22micron centrifuge filters (6,000 rpm for 5 min at 4 °C) to remove protein aggregates occurred during conjugation. The filtered protein-dye conjugates were passed through a PD-10 column (GE healthcare, USA) with a PBS to separate dye-labeled proteins from unreacted dyes. Quantitation of the protein-dye conjugation (dye:protein molar ratio) was obtained through dividing molar concentration of dye by molar concentration of protein. For this, concentration of protein was measured through Bradford protein assay, and dye concentration was determined

on the basis of maximum absorbance (675 nm) measurements by X-ma 100 spectrophotometer (Human Corporation, Korea).

Circular dichroism analysis

Circular dichroism spectra of rEgH9 and rEgH9-Cy5.5 conjugates were measured from 190 to 280 nm at 25°C using a Jasco-815 CD spectropolarimeter (Jasco, Japan). The path length of the quartz cuvettes used for rEgH9 and rEgH9-Cy5.5 conjugates is 0.5 mm. All samples were diluted in PBS (pH 7.4) at a concentration of 0.2 mg/ml.

Confocal fluorescence microscopy

Cells were plated on an eight well glass slide (SPL, Korea) for 24-hour incubation in an incubator with 5% CO_2 at 37 °C. Fluorescence-labeled proteins (10 μg/ml) diluted in a serum-free DMEM medium (Welgene, Korea) were treated for 3 hours. After washing three times each wells with DPBS (Dulbesco's PBS without calcium and magnesium, Gibco, USA), Hoechst 33342 (Thermo scientific, USA) staining was performed to stain the nuclei. After washing three times with DPBS, the cells were fixed using 4% paraformaldehyde at room temperature for 20 min in the dark. Fluorescence images of the resulting cells were obtained using a LSM880 confocal microscope (Carl Zeiss, Germany) at 400x magnification.

Flow cytometry analysis

Selected cells were stained with 100 μg/ml of repebody-Cy5.5 conjugates diluted in a FACS buffer (DPBS containing 3% FBS) at 4°C for 30 min in the dark. After staining the cells, washing steps were performed twice (1,500 rpm for 5 min) with FACS buffer. Cellular fluorescence of 1 x 10^4 cells was analyzed using a LSRFortessa™ X-20 flow cytometer (BD biosciences).

Western blot analysis

Cells (A431, HCC827, MDA-MB-468, and MCF7) were lysed in protein extraction solution and the total protein samples (20 μg) were loaded onto 8% SDS-PAGE, transferred to a polyvinylidene fluoride membranes. After blocking with a blocking buffer (PBS containing 0.05 % Tween-20 and 5% skim milk), the membrane was incubated with primary rabbit anti-EGFR antibody (Sino Biological, China), and anti-tubulin antibody overnight at 4°C. The membrane was washed with PBST (PBS containing 0.05 % Tween-20) and incubated with

HRP-conjugated secondary antibody (Millipore, USA) for 1 hour at room temperature. Immunoreactive protein bands were detected with ECL detection kit (Thermo scientific, USA) in the dark. For western blot of tumor, frozen tumor samples were grinded using liquid nitrogen-cooled mortar and lysed in RIPA Buffer (Sigma-Aldrich). Lysates were isolated by centrifugation at 15,000 rpm for 20 min at 4°C. Following steps are the same as described above.

Mouse experiment

Five-week-old BALB/c-nude female mice (Nara-Biotec, Korea) were maintained under controlled conditions of temperature (23 ± 2 °C), humidity (55 ± 5%), and light (12 h light/dark cycle) at the Korea Basic Science Institute (KBSI) and had access to food and water *ad libitum*. All animal experiments were approved by the Institutional Animal Care and Use Committee at KBSI. For the mouse xenograft model, 1×10^6 cells of A431 were subcutaneously implanted and tumor size was calculated using a formula for hemi-ellipsoid (volume = 0.5236 × length × width × height) using calipers. When the tumor size reached approximately 80 mm^3, 5 mg/kg of the repebodies was intravenously injected.

Immunofluorescence studies

Tumors were harvested 6 h after repebody injections and prepared as frozen sections. Sections on slides were fixed using 4% PFA (DaeJung Chemicals, Korea) for 30 min and incubated with anti-CD31 (rabbit anti-human CD31 monoclonal antibodies; Thermo Fisher Scientific, USA) for 2 h at room temperature. Secondary antibodies were anti-rabbit Alexa 488 (Life Technologies, USA), which were incubated for 45 min at room temperature. After washing with PBS, sections were counterstained with Hoechst 33342 (Sigma-Aldrich) at 10 μg/mL for 10 min at room temperature and examined using LSM 780 Zeiss Confocal Laser Microscope (Zeiss, Germany). Digital images were processed using the ZEN 2010 software.

Whole-body biodistribution of repebodies

Tumor-bearing mice were administered with Cy5.5 dye-tagged repebodies through tail vein injections and imaged using the VISQUE InVivo Smart LF bio-imaging system (VIEWORKS) with an excitation wavelength of 630-680 nm and an emission wavelength of 690-740 nm. The mean radiant efficiency in the region of interest was processed using the Clevue software.

Statistical analysis

Statistical comparisons of the data sets were performed by one-way ANOVA with Tukey correction using Prism software (Version 8; GraphPad Inc.). Data were considered statistically significant when $P < 0.05$.

Supplemental References

Lee, S.C., Park, K., Han, J., Lee, J.J., Kim, H.J., Hong, S., Heu, W., Kim, Y.J., Ha, J.S., Lee, S.G., et al. (2012). Design of a binding scaffold based on variable lymphocyte receptors of jawless vertebrates by module engineering. Proc Natl Acad Sci U S A *109*, 3299–3304.
